# Observing the Forest Canopy with a New Ultra-Violet Compact Airborne Lidar

**DOI:** 10.3390/s100807386

**Published:** 2010-08-06

**Authors:** Juan Cuesta, Patrick Chazette, Tristan Allouis, Pierre H. Flamant, Sylvie Durrieu, Joseph Sanak, Pascal Genau, Dominique Guyon, Denis Loustau, Cyrille Flamant

**Affiliations:** 1 Laboratoire de Météorologie Dynamique–IPSL/École Polytechnique, 91128 Palaiseau, France; E-Mail: flamant@lmd.polytechnique.fr; 2 Laboratoire Atmosphères, Milieux, Observations Spatiales–IPSL/75252 Paris, France; E-Mails: pascal.genau@latmos.ipsl.fr (P.G.); cyrille.flamant@latmos.ipsl.fr (C.F.); 3 Laboratoire des Sciences du Climat et l’Environnement–IPSL/91191 Saclay, France; E-Mails: patrick.chazette@lsce.ipsl.fr (P.C.); joseph.sanak@cea.fr (J.S.); 4 UMR TETIS, Cemagref/34196 Montpellier, France; E-Mails: tristan.allouis@teledetection.fr (T.A.); sylvie@teledetection.fr (S.D.); 5 INRA, UR1263 EPHYSE, F-33140 Villenave d’Ornon, France; E-Mails: guyon@bordeaux.inra.fr (D.G.); denis.loustau@pierroton.inra.fr (D.L.)

**Keywords:** vegetation active remote sensing, laser, canopy lidar, airborne lidar, ultra-violet emission, ultra-light airplane

## Abstract

We have developed a new airborne UV lidar for the forest canopy and deployed it in the Landes forest (France). It is the first one that: (i) operates at 355 nm for emitting energetic pulses of 16 mJ at 20 Hz while fulfilling eye-safety regulations and (ii) is flown onboard an ultra-light airplane for enhanced flight flexibility. Laser footprints at ground level were 2.4 m wide for a flying altitude of 300 m. Three test areas of ∼500 × 500 m^2^ with Maritime pines of different ages were investigated. We used a threshold method adapted for this lidar to accurately extract from its waveforms detailed forest canopy vertical structure: canopy top, tree crown base and undergrowth heights. Good detection sensitivity enabled the observation of ground returns underneath the trees. Statistical and one-to-one comparisons with ground measurements by field foresters indicated a mean absolute accuracy of ∼1 m. Sensitivity tests on detection threshold showed the importance of signal to noise ratio and footprint size for a proper detection of the canopy vertical structure. This UV-lidar is intended for future innovative applications of simultaneous observation of forest canopy, laser-induced vegetation fluorescence and atmospheric aerosols.

## 1. Introduction

An in-depth knowledge of forest ecosystem functioning is essential for sustainable management of forest resources (e.g., [1]). A detailed description of forest vegetation 3D structure is required to provide relevant information on biodiversity, available biomass and stand growth rate. Such information enables the evaluation of the efficiency of management practices and the vulnerability to natural risks (e.g., forest fires, storms, insect epidemics, *etc*). It is also highly valuable information for assessing the role of forest biomass in climate mitigation (e.g., [2]): e.g., stands of young growing trees act as efficient atmospheric CO_2_ sinks (e.g., [3]). Moreover, forest structural characterization is useful for studies concerning the role of forests in air quality: e.g., air filtering of atmospheric pollutants [4,5] and emissions of volatile organic compounds (e.g., [6]).

These environmental and socio-economical issues require extensive and accurate characterizations of forest vegetation 3D structure. In this context, the Full Waveform Technique for canopy lidars (e.g., [7]) is suitable to fulfill such a task. Typically, the first and last significant lidar returns indicate the canopy top height and ground (e.g., [8,9]). Tree height estimates and vegetation profiles by lidar can be used to assess indirectly the stand volume and carbon stock of forest (e.g., [10]). Currently, national and local agencies contract commercial companies to operate airborne canopy lidar for forest inventory. Additionally, the relevance of lidar for global forest canopy survey has been recently demonstrated by using 60 m wide footprint observations of ICES at spaceborne lidar [11]. Further improvements require the support of research airborne canopy lidar for optimizing footprint size, scanning and line-of-sight, range resolution and probing wavelength.

**Figure 1.**
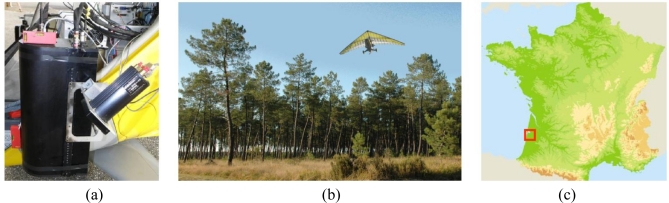
**(a)** The LAUVAC (Lidar Aéroporté UltraViolet pour l’Atmosphère et la Canopée forestière) canopy lidar on board the ultra light airplane. **(b)** Ultralight airplane flying over the Landes forest. **(c)** Location of the Landes forest (red square) in France.

We propose using the compact ultra-violet airborne LAUVAC lidar (Lidar Aéroporté Ultra-Violet pour l’Atmosphère et la Canopée forestière, see Figure 1(a)) that has been designed as a multi-purpose system for research activities with high flexibility in terms of instrumental parameters (telescope field of view, laser divergence and emitted energy). It was developed by the Commissariat à l’Énergie Atomique (CEA) and the Centre National de la Recherche Scientifique (CNRS) to be used for atmospheric pollution [12,13] and climate studies [14]. Recently, it was adapted for airborne operation by CEA with the support of Centre National d’Etudes Spatiales (CNES) to study a Maritime pine (*Pinus pinaster*) forest in the Landes region (France, Figure 1(c)) in the framework of a program initiated by the Institut Pierre Simon Laplace (IPSL) and the Centre National du Machinisme Agricole, du Génie Rural, des Eaux et Forêts (CEMAGREF).

The LAUVAC is attractive for quick and relatively inexpensive deployment onboard an ultra-light airplane (ULA, see Figure 1(b)). To our knowledge, it is the first canopy lidar operating in the UV domain (355 nm). This enables emission of energetic laser pulses (*i.e.*, 16 mJ) under eye-safe conditions, since UV radiation (<380 nm) is absorbed by the eye cornea and the crystalline before reaching the retina. By comparison, lidars operating in the visible and near IR (<1.2 μm) are eye-safe only when they emit ∼100 less energy onto the same surface (e.g., commercial systems operating at 1,064 nm typically use 0.2 mJ pulses). This is due to the fact that such radiation is focused to an intensity on the retina 100,000 times higher than at the point where the laser beam enters the eye.

With respect to canopy applications, the vegetation reflectivity in the UV is lower by a factor of ∼10 than the reflectivity in the near IR, so the ambiguity of the lidar return time of flight induced by multiple-reflectivity in the tree foliage is expected to be smaller (for equal emitted energy, [15]). A footprint of 2 to 4 m (see Section 2) is intermediate between narrow footprint (0.15 m) used by commercial lidars for very accurate urban building description and other dedicated research airborne canopy lidars (∼10 m, e.g., [8,9]) and spaceborne lidar like ICESat (60 m, e.g., [11]).

The purpose of the present paper is to demonstrate the performance of a UV lidar for canopy characterization. First, we use the full waveform canopy data to reconstruct the 3D structure of delineated and characterized forest areas in the Landes forest (one of these areas is used for long term monitoring and research activities by Institut National pour la Recherche Agronomique). Second, we validate the lidar retrievals using *in situ* measurements taken from the ground by field foresters (e.g., [16]). Section 2 presents the lidar instrumentation and the test areas in the Landes forest where the field experiments were conducted. Section 3 introduces the methodology used to retrieve: canopy tops, apparent tree tops, apparent crown bases and undergrowth heights. Section 4 shows the lidar-derived canopy structural parameters for each of the test areas and provides with comparisons to *in situ* measurements. The performance assessment is conducted in two steps by: (i) a statistical comparison between canopy lidar and *in situ* observations (of a forest stand and several sample plots), and then (ii) a one-to-one comparison between the canopy lidar and *in situ* observations. A summary and perspectives for future work are given in Section 5.

## 2. Experimental Setup

### 2.1. Canopy Lidar Payload Onboard an ULA

The UV lidar onboard the ULA looks downward close to nadir (see Figure 1). It is built around a compact 355 nm tripled Nd-YAG laser that provides 16 mJ with 5 ns pulse duration at 20 Hz pulse repetition frequency (see Table 1). Eye safety conditions are met at the exit of the optical head (Figure 1(a)). The full waveform lidar signal is digitized at a 100 MHz sampling frequency or 1.5 m sampling resolution along the lidar line-of-sight. The lidar signal acquisitions are recorded during 1 s (20 consecutive shots) and then the data are stored on an on-board portable computer for 1 s. The shot to shot separation at ground is about 1 m for an ULA horizontal velocity of 20 m s^−1^. The laser footprint at ground level has a nearly circular shape of 2.4 m diameter for a 300 m flying altitude above ground level (agl) and nadir pointing. Depending on the ULA attitude while in the air, the successive laser footprints move randomly within 10 to 40 m around the ULA ground track (see Section 3.4). A global positioning system (GPS) tracks the ULA position with an accuracy of 5 m. The three angles (yaw, pitch and roll) between the actual lidar line-of-sight and nadir direction are recorded with a 0.5° accuracy (*i.e.*, 2.6 m at the ground from an altitude of 300 agl) to locate the lidar shot. Considering the root-mean-square errors of the GPS and the three line-of-sight angles, we estimate the overall absolute precision of ∼7 m.

The ULA carrying the LAUVAC system is a Tanarg 912-S manufactured by Air Création (http://www.aircreation.fr). It is highly maneuverable (see Table 1) and it only requires a 200 m long path for take off and landing. The maximal payload is 250 kg (including the pilot) and the flying endurance is 3 to 4 hours (here the canopy flights lasted for 2 hours). The lidar optical head is installed on an axis perpendicular to the ULA displacement direction so as to change the pointing angle before the flight.

### 2.2. Test Areas

**Figure 2.**
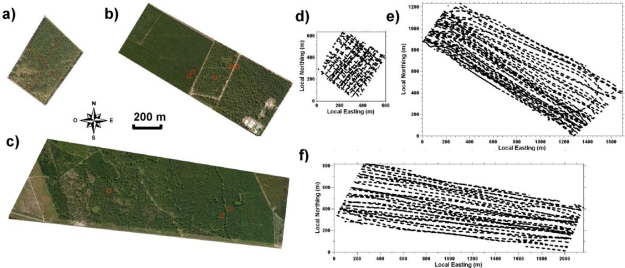
Satellite views of the three forest study areas (panels a, b and c; from Google Maps) and examples of trajectories of the airborne lidar overflying each of the areas (panels d, e and f). Bray #B area is shown in panels (a) and (d). Mimizan #M1 in panels (b) and (e). Mimizan #M2 in panels (c) and (f). In panel **(a)**, red dashed lines depict the sub-areas analyzed in Section 4.2. In panels **(b)** and **(c)**, red squares are the sample plots considered in Section 4.3 and Section 4.4. In panels **(d)**, **(e)** and **(f)**, the coordinate origins are located at 44.71°N 0.77°W, 44.15°N 1.18°W and 44.18°N 1.17°W, respectively.

The field experiments were conducted in September 2008 over three test areas located in the Landes forest region near the towns of Bray (test area #B) and Mimizan in southwestern France (two test areas referenced as #M1 and #M2; see satellite pictures in Figure 2(a,b,c)). The selected areas are mainly flat and populated with stands of Maritime pines (see example in Figure 1(b)) spanning a range of ages and either planted or naturally regenerated. These trees may reach a maximum height of 25 m in their mature stage, mostly corresponding to a trunk without living branches roughly 15 m high and a crown with branches and needles of nearly 10 m high and 5 to 8 m wide (e.g., [16]). The Bray area (#B, Figure 2(a)) is populated by regularly planted 28-year-old trees with a total tree height (*TTH*) of 20–25 m. The Mimizan #M1 area (Figure 2(b)) is composed of two sub-areas: the southeastern half of the area is composed of a 55-year-old naturally regenerated stand with *TTH* around 20 m and a northwestern sub-area of young 10-year-old plantation of 10 m height. The Mimizan #M2 area (Figure 2(c)) has several stands of different tree types and heights. From west to east, a quarter of the area is covered by sparse trees and bare ground, a quarter with semi-natural 50-year-old trees with *TTH* of 20–22 m, a quarter with planted younger 35-year-old slightly higher trees (with *TTH* ∼23 m) and a quarter of 19-year-old trees with *TTH* of 15–16 m. Density and height of undergrowth vegetation vary according to the species composition and the management of the stands (particularly dense for young tree sectors and up to ∼2 m for fern patches in the #M2 area).

Most of the airborne lidar measurements were conducted with the lidar beam pointing at nadir at an approximately constant altitude agl (300 m ± 30 m). The ULA sampled the test areas according to a grid pattern for Bray (#B) or longitudinal pattern for Mimizan #M1 and #M2 (see Figure 2(d,e,f)). For two-hour flights, the resulting spacing between lidar tracks was in average 6 to 7 m. To test the sensitivity of the lidar system retrievals to ULA altitude and beam pointing, the Bray #B area was overflown at 500 m agl with the lidar looking at nadir (*i.e.*, it results in a 4 m laser footprint) and at 300 m agl with a beam at 20° from nadir (see Section 4.2).

## 3. Processing Lidar Full Waveforms for Forest Structure Parameter Retrieval

For retrieving forest structure parameters, full waveforms acquired by LAUVAC are processed according to the following steps:

- We use all lidar signals for a desired flying height (*i.e.*, ∼300 m) and a pointing angle near nadir (*i.e.*, <10° from nadir). Provided high enough signal-to-noise ratio (*SNR*) for LAUVAC waveforms, no filtering nor pre-processing for increasing the ranging accuracy is needed (see Section 3.1).
- For each waveform, we subtract the continuous component of noise (*i.e.*, background noise from solar luminance and atmospheric scattering), which is estimated as the mean signal over 150 m above the canopy.
- Then, we retrieve relevant forest structure parameters (see Section 3.2) using a threshold method. We calculate a threshold value for each flight by distinguishing the histograms of canopy return signals (for the forest canopy and ground) and noise fluctuations (see Section 3.3).
- We determine the ground location for each waveform, verifying shot-to-shot consistency, and then we derive the structural parameters of the canopy above (see Section 3.4).

### 3.1. LAUVAC Lidar Signals

Laser emission of energetic pulses (16 mJ) and the relatively low flying heights (∼300 m) of LAUVAC provide much higher canopy and ground return signals than shot noise fluctuations, *i.e.*, typical *SNR* ranges from 300 to 800. Within the length of the laser pulses (1.5 m), only one signal sample is available and we expect no correlation induced by the laser pulse width between consecutive waveform points. Considering these remarks, we presume that no pre-processing is needed for deconvolving the laser pulse shape (e.g., [17]) nor improving the accuracy for detecting canopy signal peaks (e.g., [18]), as widely done for other systems [17,18]. In order to test these assumptions, we performed numerical simulations of waveform decomposition into Gaussian functions (following [18]) for different instrumental configurations.

**Figure 3.**
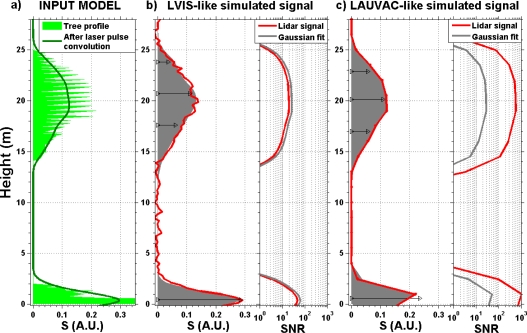
**(a)** Input model for numerical simulation of a synthetic tree profile before (green shade) and after (dark green line) convolution by a Gaussian laser pulse shape (1.5 m of half-width) expressed in lidar power signal *S* in arbitrary units (A.U.). **(b)** Lidar waveform (red line on left panel) acquired by a system like LVIS (see [18], with 0.3 m vertical resolution and *SNR* on the right panel) and Gaussian fit waveform (gray shade) obtained by fitting four Gaussian functions (with locations and amplitudes indicated by black lines with triangles). **(c)** Same as (b), but for a LAUVAC-like system (1.5 m vertical resolution and *SNR* up to 800).

First, we considered a power signal *S* by convolving a synthetic tree attenuated backscatter profile by a Gaussian laser pulse shape of 1.5 m of length (Figure 3(a)). We sampled *S* and added random noise according to two lidar configurations: (i) 1.5 m vertical resolution and *SNR* up to 800 as for LAUVAC and (ii) 0.3 m vertical resolution and *SNR* from 20 to 50 as obtained by other systems such as LVIS (see e.g., [18]). For the LVIS-like configuration (Figure 3(b)), spurious fluctuations are removed and the actual *SNR* is increased by ∼10 in the waveform retrieved by fitting four Gaussian functions (gray shade). However, in the case of the LAUVAC-like waveform (Figure 3(c)), no significant difference is apparent between the waveforms with (gray shade) and without (red line) the Gaussian fitting procedure. Moreover, the limited number of points for constraining the Gaussian curves induces errors greater than the random noise fluctuations, which degrade the *SNR* after the fitting procedure (gray line in right panel of Figure 3(c)). Besides, as the central point of the Gaussian functions (black small triangles in Figure 3(b,c)) indicates the center of the canopy reflecting surfaces [18], no additional information is provided about tops and bases of tree crowns (which are retrieved in the present paper, see Section 3.2). For these previous reasons, we have chosen a threshold method as describe hereafter.

### 3.2. Canopy Structure Parameters

**Figure 4.**
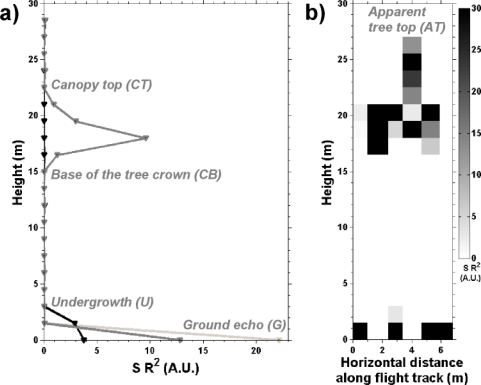
**(a)** Three examples of lidar profiles: for bare ground (in light gray), ground with undergrowth vegetation (in black) and a single maritime pine with the ground return below (in dark gray). *SR^2^* is the range-corrected lidar signal (in A.U.) (e.g., [19]). The structural parameters *AT, CT*, *CB* and *U* (in gray and italics) are analyzed in Sections 4. **(b)** Example of 6 consecutive lidar profiles describing the 2D structure of one single tree crown and undergrowth vegetation.

Four significant ranges are extracted from the full waveform lidar signals (see Figure 4(a)): (i) the ground range (*G*) as indicated by the last return, (ii) the canopy top height (*CT*) by subtracting *G* from the first return range, (iii) the crown base height (*CB*) as the last return after propagation in the tree foliage and (iv) the undergrowth height (*U*) when the ground return is broader than the 1.5 m sampling resolution (see Figure 4(a)). Then, we derive an apparent tree top height (*AT*) from the *CT* distribution: *AT* are the local maxima of the 2D horizontal distribution of *CT*, as adjacent lidar profiles sample in detail the shape of tree crowns (see Figure 4(b)). Among all local maxima, we only keep the highest ones within a proximity radius equivalent to the horizontal size of the tree crowns. In practice, a 2.5 m radius is used, which is the average of the *in situ* measurements performed in the test areas (for trees from 10 to 55 years old).

### 3.3. Threshold Method

We use a threshold method to estimate automatically *G*, *CT*, *CB* and *U* from each single-shot lidar signal profile. Lidar power signal *S* is used to avoid multiplication of noise by the squared range. Reliable vegetation canopy signals are identified as all return signals exceeding a detection threshold *t* at ranges above *G* (taken as the maximum value of the ground signal peak). A false alarm occurs when the signal noise exceeds *t* and no detection occurs when the return signal of the canopy top *CT* (or *CB*, *U*, *G*) is below *t*. The value of *t* is chosen to minimize the total probability of error *p_T_* on a given profile. This probability depends on *p_F_* and *p_N_*, respectively the probabilities of false alarm and no detection. Their product *p_F_ p_N_* is the probability that for a profile both errors occur simultaneously. To calculate *p_T_*, we subtract *p_F_ p_N_* from the sum *p_F_ + p_N_* in order not to account for it twice. Hence we write *p_T_* as (e.g., [20,21]):

(1) $$p_{T}=p_{F}+p_{N}-p_{F}\;p_{N}$$

To estimate *p_F_* and *p_N_*, we first use the lidar signal histogram in the detection range to identify and calculate the noise and canopy signals probability functions after normalization *i.e.*, *F_N_* (S) and *F_S_* (*S*), respectively.

**Figure 5.**
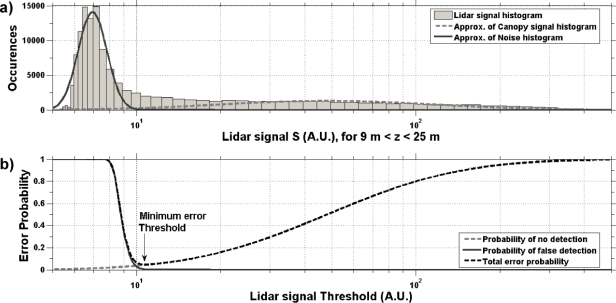
**(a)** Histogram of lidar power signals *S* (in arbitrary units) between 9 and 25 m above the ground return. Log-normal distributions are used to depict the probability density functions of the canopy (dashed gray line) and noise (plain dark gray line) contributions to *S*. To avoid truncation of negative values, an offset of 3 times the standard deviation of noise is added to *S*. **(b)** Probabilities of no detection (dashed gray line), false detection (plain dark gray line) and total error (dashed black line) according to the probability distributions derived from panel (a).

Figure 5(a) shows an example of a histogram of lidar signal *S* (here between 9 and 25 m above the ground) which is modeled as the sum of two log-normal functions that account respectively for the contributions of noise and canopy returns. We first identify the noise histogram *F_N_* (S) as the lower peak of the signal histogram and we fit a Gaussian curve by a classical method (e.g., [18]). We obtain the probability function of the useful signal *F_S_* (*S*) from the remaining part of the histogram. Then, we calculate *p_F_* and *p_N_* as (e.g., [20]):

(2) $$p_{N}(t)=1-F_{S}(t)$$

(3) $$p_{F}(t)=1-{\left(1-F_{F}(t)\right)}^{M}$$

where *M* is the number of independent points within the detection range. According to the example showed in Figure 5(b), canopy detections are expected in 95% of the cases (or equivalently 5% probability of error) when we use the optimum *t* value (which minimizes *p_T_*). For this example, the mean ground return is approximately a factor 4 higher than the mean canopy returns (not shown). Then, the ground return probability distribution is less overlapped with the noise probability distributions and thus less sensitive to the choice of *t* (the minimum of *p_T_* is mostly flat over a wider range of *t* values).

### 3.4. Analysis of Horizontal Transects of Lidar Profiles

**Figure 6.**
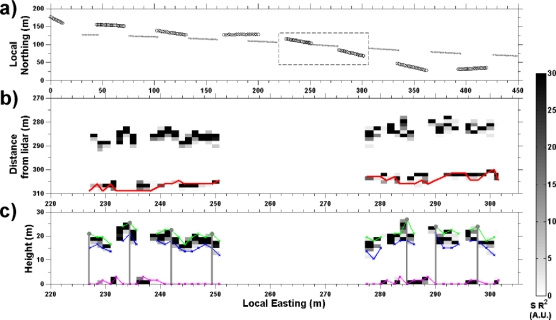
Horizontal transect of lidar profiles, example from Mimizan #M2. **(a)** ULA ground track (light gray dots, the void areas correspond to data acquisition interruptions for storage) and corresponding laser footprints at the ground (black circles). **(b)** 2D suite of lidar profiles (dashed rectangle in panel a) as a function of distance from the lidar (gray shading) and the corresponding ground return detected for each profile (red line). **(c)** 2D suite of lidar profiles (gray shading) with respect to height above the ground after correction for angular effects and corresponding detections of *CT* (green), *AT* (gray stems), *CB* (blue) and *U* (fuchsia).

A horizontal transect of lidar profiles and the corresponding detected parameters are shown in Figure 6. Following the ULA attitude during flight, the lidar footprint (black circles in Figure 6(a)) moves around the flight track (gray dots).

Due to the vertical displacement of the ULA, we observe a progressive variation in range between the lidar and the ground return (detected in most profiles and displayed in red in Figure 6(b)). After the shot-to-shot ground detection, the coherence between adjacent information is verified. If they differ by more than 5 m (∼5% of cases), the ground range is determined by interpolation between the two closest lidar profiles. Lidar profiles are then projected in the vertical direction (gray shading in Figure 6(c)) according to the ULA attitude (*i.e.*, pitch and roll). With a typical 5° deviation from nadir, the correction is negligible (Figure 6(c)). Canopy parameters *CT*, *CB* and *U* are then retrieved and *AT* is obtained from the *CT* horizontal distribution (Figure 6(c)).

## 4. Results

In this section, we present the canopy 3D overall structure as retrieved by the canopy lidar (see Section 4.1). Then, we show statistical comparisons between the lidar and *in situ* information for one of the analyzed test areas (a stand of ∼500 × 500 m^2^, see Section 4.2) and for several sample plots of the other two areas with trees of different ages (8 plots of 30 × 30 m^2^, see Section 4.3). Finally, we present one-to-one comparisons between lidar and *in situ* measurements (see Section 4.4).

### 4.1. Overall 3D Canopy Structure

**Figure 7.**
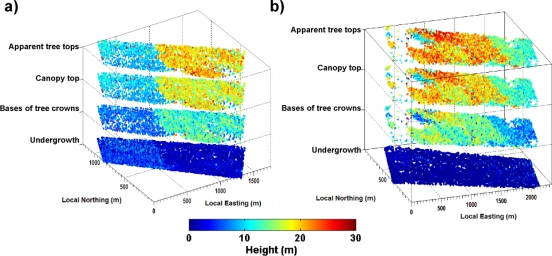
Vegetation distribution of the Mimizan **(a)** #M1 and **(b)** #M2 test areas as retrieved from canopy lidar measurements. The color code indicates the value (in m) of the four parameters *AT*, *CT*, *CB* and *U* displayed as at 4 level surfaces.

Figure 7 displays the horizontal distribution of *AT*, *CT, CB* and *U* for Mimizan #M1 (Figure 7(a)) and #M2 (Figure 7(b)), as a function of local easting (LE) and local northing (LN).

The discontinuities between tall (*AT* ≥18 m) and short (*AT* ≤10 m) trees are clearly marked (at ∼500 m LE for #M1 and ∼1700 m LE for #M2) as shown by the color code transitions e.g., from light-blue/green to orange/red for *CT*. This agrees with the corresponding distribution of mature and young trees within the test areas (see Section 2.2). Figure 7(b) shows a more heterogeneous height distribution for mature trees of #M2 (from 500–1,500 m LE). This is related to differences in height between taller planted trees (500–1,000 m LE 0–500 m LN) and relatively shorter naturally regenerated trees (500–1,000 m LE 500–1,000 LN and also 1,000–1,500 LE). As expected, higher (respectively smaller) undergrowth is associated with the younger (respectively older) forest stand. Clear void zones without trees (see Figure 2(b,c)) are correctly identified (*i.e.*, no canopy top and tree crown base information extracted from lidar signals; e.g., ∼1,200 m LE ∼0 m LN in Figure 7(a) and 100–300 m LE in Figure 7(b)).

**Figure 8.**
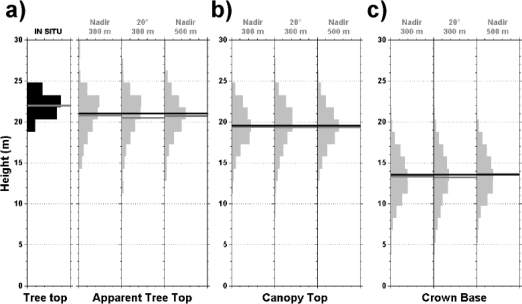
Histograms of the structural parameters for Bray #B as measured *in situ* (*TTH* in panel (a) and retrieved by canopy lidar (*AT* in panel a, *CT* in panel b and *CB* in panel c). Three different flying configurations are considered: (i) nadir pointing and (ii) pointing at 20° from nadir when flying at 300 m agl, as well as (iii) nadir pointing from an altitude of 500 m agl. All histograms (black or gray areas) are normalized and displayed from 0 to 0.5 in the horizontal axis with the mean/median values (gray/black line). All lidar data were processed using the optimum threshold.

Lidar retrievals for #B present good coherence of mostly homogeneous canopy top heights (not shown on the figures) for this uniformly planted forest stand. Mean *CT* and *AT* (see histograms in Figure 8(a,b)) of 19.5 m and 21.2 m, respectively, are observed. Tree crown bases are located on average 7.5 m below the tree tops. As confirmed by *in situ* information, only sparse undergrowth vegetation in the first meter above the ground was observed for #B.

### 4.2. Statistical Comparison with *in situ* Measurements of a Forest Stand

We compare histograms of *in situ*-measured total tree heights *TTH* of 100 trees selected at random in the Bray #B area (1 forest stand) with *AT* retrieved by lidar and checked the consistency of the lidar retrieval for three different flight configurations: (i) nadir pointing, (ii) pointing at 20° from nadir pointing flying at 300 m agl as well as (iii) nadir pointing at 500 m agl. Tree top height histograms (Figure 8(a)) from *in situ* operator and lidar show a very good agreement (particularly for a flight altitude of 300 m agl and for *AT* ≥19 m).

Apparent tree top heights retrieved by lidar between 15 m and 19 m do not correspond to actual tree tops, as shown by comparison to *in situ* measurements. In those cases (∼20%), the laser footprints sample the side and not the top of the trees (notice that false alarms are only 5% of the cases). Such effect produces a 1 m underestimation of mean tree top height (see Figure 8(a) and Table 2), which is smaller than the actual lidar sampling resolution of 1.5 m.

Provided that a proper detection threshold *t* is selected (see Section 3.3), the results on Figure 8 show good consistency between the canopy lidar retrievals for the different flight configurations. The resulting histograms are almost identical for all the parameters of interest (*AT*, *CT* and *CB*). Table 2 shows in more details the results, and especially for the 500 m agl data when they are processed using the same threshold as for the 300 m flights indicated as “1.5*t*” (it is 50% higher than the optimum value for the 500 m flight data using Equation 1 to 3).

For such a non-optimal threshold value, it results in 1.5 m underestimation of the canopy top (and *AT*). So, for too high threshold value the weak signal that marks the canopy top transition may be missed. However, this bias cancels when the optimum threshold is used (see Table 2 and Figure 8). For the crown bases heights (and ground returns), a robust detection is indicated by almost identical results for the three flights (Figure 8(c)), even using different thresholds (see Table 2). This is probably due to a higher density of the scattering volume (foliage and branches) for the crown bases.

For verification consistency at smaller scales, we have analyzed Bray #B area divided into 4 quadrants of equal area (see Figure 2(a)). Table 2 shows that similar results were also obtained for *AT*, *CT* and *CB* for each of these quadrants (with differences of about 1 m between quadrants) both in the comparisons between different lidar retrievals (different configurations and thresholds) and with *in situ* measurements.

### 4.3. Statistical Comparisons with *in situ* Measurements of Several Sample Plots

Additional statistical comparisons between lidar and *in situ* information were conducted for smaller sample plots (of 30 × 30 m^2^) representative of different stands within the test areas #M1 (five plots #M1A to #M1F, see location in Figure 2(b)) and #M2 (three plots #M2A to #M2C, see Figure 2(c)). All sample plot comparisons are summarized in Table 3.

We compare both tree top and crown base heights as measured *in situ* (*TTH* and *CB_IS_*, respectively) and by airborne canopy lidar (*AT* and *CB* respectively). Good consistency, within 0.5 to 1 m, is verified for tree top and crown base average heights for mature trees plots (*i.e.*, #M1C, #M1D, #M1E and #M2A). Young tree sample plot comparisons show as well a good accuracy in the order of 1 m for the tree top retrievals. However, the crown bases estimations by lidar present a 2 m overestimation (#M1A and #M1B in Table 3). This could be related to a limited penetration of the laser beam into denser foliage of young trees.

### 4.4. One-to-One Comparisons with *in situ* Measurements

**Figure 9.**
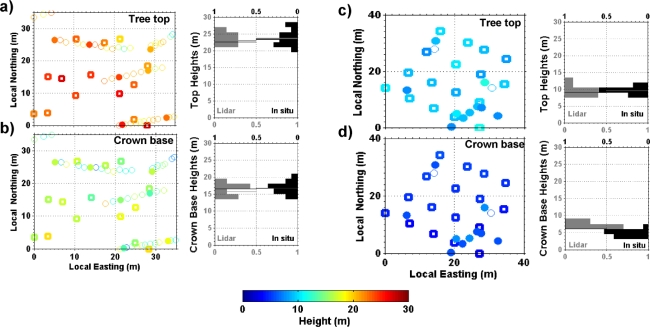
Comparison of structural parameters as retrieved by canopy lidar and *in situ* measurements for two sub-samples of Mimizan #M2B (panels a and b, with 14 trees) and #M1B (panels c and d, with 17 trees) study areas. Panels **(a)** and **(c)**: we compare tree top height retrieved from *in situ* (*TTH*/open squares) and canopy lidar (*AT*/filled circles and *CT*/open circles) observations with respect to LE and LN (left panels). We also provide histograms (right panels) of tree top height from *in situ* (black) and from canopy lidar (gray) measurements with the corresponding mean/median values (black line/gray line). Panels **(b)** and **(d)**: same as (a) and (c), but for crown base height *CB*.

Given the tree population and distribution densities in Mimizan #M1 and #M2 tests areas, the geo-location of the canopy lidar profiles were accurate enough to draw unambiguous one-to-one correlation with *in situ* measurements. This sub section presents the relevant comparisons between lidar and *in situ* observations. Two examples of coincidences for 14 and 17 trees, respectively, are displayed on Figures 9(a,b) (#M2B) and 9(c,d) (#M1B) for illustration.

Figure 9 shows a comparison of tree top and crown base heights as measured *in situ* (open bold squares) and by airborne canopy lidar (plain circles). Within the 7 m uncertainty on lidar footprint location at ground, very good agreement is found for measurements that are likely to correspond to the same trees in Figure 9(a,b) (e.g., for geo-location at 28 m LE, 18 m LN; 22 m LE, 13 m LN; and 13 m LE 26 m LN). Good measurements consistency is found for the histograms of tree top and crown base height measurements (to the right of Figure 9(a,b)).

Figure 9(c) indicates that for a young tree sample plot (#M2B) we obtain good matches (±1 m) for measurements of tree tops (e.g., at 15 m LE, 33 LN; 28 m LE, 8 LN; 21 m LE 3 m LN), but an overestimation of ∼2 m for the crown base (see histogram to the right of Figure 9(d); as mentioned from Table 3).

## 5. Summary and Perspectives

The results of field experiments conducted in the Landes Maritime pine forest have demonstrated the capability of the new UV canopy lidar LAUVAC to accurately measure detailed forest canopy vertical structure. The LAUVAC observations characterized the spatial distribution of tree tops and crowns with a 1.5 m height relative accuracy (due to lidar digitization unit) and horizontal geo-location of 7 m (due to lidar pointing and ULA instrumentation). The 2.4 m footprint and high energy emitted per pulse result in good detection sensitivity and enables the observation of ground returns underneath the trees. The results demonstrate an overall consistency of the canopy features (e.g., changes in tree heights with age, horizontal homogeneity) and retrieval of the 3D canopy structure. Comparisons with *in situ* measurements indicated that the current LAUVAC measurements present an absolute accuracy of about 1 m. Sensitivity tests on detection threshold have shown the importance of signal to noise ratio and footprint size for a proper detection of the canopy vertical structure.

Future work will be conducted using LAUVAC with co-localized *in situ* observations (*i.e.*, for biomass) and onboard observations with the same ULA by twin 3-bands cameras in the UV, visible and near IR. We will conduct studies on both: (i) forest canopy light reflectivity and (ii) biomass quantification using LAUVAC profiles, after correction of signal attenuation by foliage partial occlusion and calibration with ground return intensity (mostly observed) and atmospheric backscattering (significant in the UV). Improvement of LAUVAC capability for canopy applications will aim at inferring the forest biomass and growth rate (approx. 1 m per year). To do so, the vertical resolution of the lidar will be improved to 0.375 m and a continuous data acquisition system will record all contiguous laser profiles. Forthcoming field experiences will be performed over different kinds of forest (*i.e.*, deciduous and coniferous trees).

In addition to lidar ranging capability, information on canopy health and stress can be provided by fluorescence measurements using UV laser emission (e.g., [22]). Such key information combined with passive radiometric measurements and standard foliage indices (e.g., [23]) are essential for studying the variability of biogenic emissions and their role in atmospheric photochemistry. Our goal in the near future is to develop a multi-purpose UV airborne lidar to simultaneously monitor vegetation structure and laser-induced fluorescence response as well as overlaying atmospheric aerosols for biosphere-atmosphere exchanges.

## Figures and Tables

**Table 1.** Characteristics of the canopy lidar LAUVAC onboard an ULA. LAUVAC is a prototype of the EZlidar manufactured by Leosphere^™^ (http://www.leosphere.com).

LIDAR	Laser emission wavelength: 355 nm
	Laser energy per pulse: 16 mJ (fulfilling eye-safety requirement)
	Laser pulse duration: 5 ns (1.5 m pulse length along the line-of-sight)
	Repetition rate: 20 Hz (it corresponds to 1 m horizontal spacing between canopy lidar profiles for 20 m s^−1^ ULA speed)
	Vertical resolution: 1.5 m (according to 100 MHz signal digitization and profiling at nadir)
	Laser divergence: 4 mrad (that corresponds to a 2.4 m-diameter footprint at a flying altitude of 300 m agl)
	Receiver field of view: 5 mrad
	Detector: photomultiplier tubes/analog direct detection
	Optical head dimensions: 45 × 28 × 18 cm^3^
	Weight: 9 kg (optical head) + 20 kg (electronic unit)
	Electrical supply: 12 V battery (<500 W)
Geo-referencing system	GPS: Lassen SK II by Trimble (±5 m at 1 Hz)
	Artificial horizon: Dynon Avionics (±0.5 at 1 Hz)
Ultra light Airplane	Maximal scientific payload: 120 kg
	Flight speed: 17 to 40 m/s (60 to 145 km/h)
	Endurance: 4 h at 20 m/s (3 h at 40 m/s)
	Flight altitude: between 200 m and 5.8 km agl In practice 300 m and 500 m agl for canopy flights.

**Table 2.** Statistical comparisons (in m) between *in situ* and canopy lidar observations for Bray #B test area. Results are also provided by quadrant. We indicate *in situ* (*TTH* first row) and canopy lidar (*AT, CT, CB*) measurements in mean/median values (and standard deviations). Three different flying configurations are considered: (i) nadir pointing and (ii) pointing at 20° from nadir when flying at 300 m agl, as well as (iii) nadir pointing from an altitude of 500 m agl. Data processing using a threshold 50% higher is indicated as “1.5*t*”. The number of *in situ* samples for each quadrant is 11 for NE, 0 (n/a) for NW, 26 for SW and 62 for SE.

**Structural parameter**	**Retrieval method**	**Whole area**	**NE quadrant**	**NW quadrant**	**SW quadrant**	**SE quadrant**
Tree top *TTH*	*in situ*	22.0/22.0 (1.2)	22.1/21.9 (0.9)	n/a	21.6/21.5 (1.2)	22.1/22.3 (1.2)
Apparent tree top *AT*	300 m Nadir	21.2/21.0 (4.0)	21.1/21.0 (4.1)	21.3/21.0 (3.6)	21.1/21.0 (4.6)	21.2/21.0 (3.5)
	300 m 20°	20.5/21.0 (3.4)	19.6/19.5 (3.4)	20.8/21.0 (3.6)	21.9/22.5 (2.7)	20.0/21.0 (3.5)
	500 m Nadir	20.7/21.0 (3.3)	20.9/21.0 (2.8)	20.9/21.0 (3.2)	20.5/21.0 (3.3)	20.5/21.0 (3.6)
	500 m Nadir 1.5*t*	19.9/19.5 (2.7)	20.4/21.0 (2.4)	20.3/21.0 (2.5)	19.8/19.5 (2.6)	19.5/19.5 (3.0)
Tree top difference (*TTH*-*AT*)	300 m Nadir	1.0/1.0	1.0/0.9	n/a	0.5/0.5	0.9/1.3
	300 m 20°	1.7/1.0	2.5/2.4	n/a	−0.3/−1.0	2.1/1.3
	500 m Nadir	1.5/1.0	1.2/0.9	n/a	1.1/0.5	1.6/1.3
	500 m Nadir 1.5*t*	2.3/2.5	1.7/0.9	n/a	1.8/2.0	2.6/2.8
Canopy Top *CT*	300 m Nadir	19.7/19.5 (3.1)	19.9/19.5 (3.0)	19.6/19.5 (2.9)	19.7/19.5 (3.6)	19.5/19.5 (3.1)
	300 m 20°	19.3/19.5 (3.2)	18.4/18.0 (3.3)	19.5/19.5 (3.4)	19.9/19.5 (2.9)	19.2/19.5 (2.9)
	500 m Nadir	19.3/19.5 (2.8)	19.4/19.5 (2.7)	19.5/19.5 (2.8)	19.2/19.5 (2.8)	19.2/19.5 (2.9)
	500 m Nadir 1.5*t*	18.6/18.0 (2.6)	18.8/19.5 (2.6)	18.9/19.5 (2.5)	18.5/18.0 (2.6)	18.3/18.0 (2.6)
Apparent crown base *CB*	300 m Nadir	13.5/13.5 (3.4)	13.2/13.5 (3.0)	13.7/13.5 (3.2)	13.8/13.5 (3.9)	13.0/13.5 (3.1)
	300 m 20°	13.2/13.5 (3.5)	12.5/12.0 (3.6)	13.2/13.5 (3.7)	13.7/13.5 (3.5)	13.2/13.5 (3.4)
	500 m Nadir	13.6/13.5 (3.2)	13.5/13.5 (3.1)	13.8/13.5 (3.0)	13.5/13.5 (3.1)	13.6/13.5 (3.3)
	500 m Nadir 1.5*t*	13.5/13.5 (2.9)	13.5/13.5 (2.9)	13.9/13.5 (2.7)	13.5/13.5 (2.8)	13.4/13.5 (3.0)

**Table 3.** Comparison of forest structural parameters (in m) as retrieved using the canopy lidar and *in situ* measurement. Mean/median values (and standard deviations) for each parameter within eight sub samples of approximately 30 × 30 m^2^ which are representative of each tree population. Sample locations are indicated in Figure 2(b,c) and given with respect to Figure 2(e,f) axes.

**Same plot**	**Location of plot centre**	**Tree top**			**Canopy top**	**Crown base**		
		***TTH* (*in situ*)**	***AT* (lidar)**	***TTH–AT***	***CT* (lidar)**	***CB_IS_* (*in situ*)**	***CB* (lidar)**	***CB_IS_–CB***
#M1A	580 LE 600 LN	9.1/9.2 (0.8)	9.4/9.0 (0.8)	−0.3/0.2	9.3/9.0 (0.6)	5.3/5.4 (0.6)	6.8/6.8 (0.9)	−1.5/−1.4
#M1B	615 LE 660 LN	9.5/9.4 (0.9)	8.8/9.0 (1.5)	0.7/0.4	8.7/9.0 (1.4)	4.5/4.7 (0.8)	6.5/6.0 (0.7)	−2.0/−1.3
#M1C	780 LE 610 LN	19.8/19.5 (1.1)	20.3/21.0 (1.5)	−0.5/−1.5	17.7/18.0 (2.3)	14.2/14.4 (1.5)	15.4/15.8 (2.6)	−1.2/−1.4
#M1D	930 LE 690 LN	21.9/22.1 (0.9)	21.0/21.0 (2.1)	0.9/1.1	18.0/18.0 (2.7)	15.8/15.4 (1.0)	15.4/14.3 (2.4)	0.4/1.1
#M1E	1050 LE 690 LN	21.9/21.4 (1.2)	21.8/21.8 (1.1)	0.1/−0.4	17.6/18.8 (4.4)	15.8/15.7 (0.9)	18.8/18.8 (1.1)	−3.0/−3.1
#M2A	850 LE 330 LN	20.8/20.5 (1.9)	19.7/19.5 (2.0)	1.1/1.0	19.1/19.5 (1.1)	13.8/14.3 (4.0)	13.5/13.5 (3.0)	0.3/0.8
#M2B	1630 LE 170 LN	23.6/23.4 (2.2)	22.9/22.5 (1.7)	0.7/0.9	19.7/21.0 (3.2)	16.7/16.6 (1.7)	15.6/16.5 (1.7)	1.1/0.1
#M2C	1700 LE 210 LN	16.0/16.4 (1.2)	15.4/15.0 (2.1)	0.6/1.4	14.1/15.0 (2.7)	10.3/10.2 (0.6)	10.3/9.0 (2.8)	0.0/1.2
